# Evaluation of the diagnostic accuracy and cost of different methods for the assessment of severe  anaemia in hospitalised children in Eastern Uganda

**DOI:** 10.12688/wellcomeopenres.14801.2

**Published:** 2019-03-18

**Authors:** Peter Olupot-Olupot, Natalie Prevatt, Charles Engoru, Julius Nteziyaremye, Denis Amorut, Martin Chebet, Tonny Senyondo, Paul Ongodia, Carolyne M. Ndila, Thomas N. Williams, Kathryn Maitland

**Affiliations:** 1 Faculty of Medicine, Imperial College London, London, W2 1PG, UK; 2 Department of Paediatric, Busitema University Faculty of Health Sciences, Mbale Campus, Uganda; 3 Mbale Clinical Research Institute, Mbale, Uganda; 4 London School of Tropical Medicine and Hygiene, London, UK; 5 Department of Paediatrics, Soroti Regional Referral Hospital, Soroti, Uganda; 6 Department of Paediatrics, Mbale Regional Referral Hospital, Mbale, Uganda; 7 KEMRI Wellcome Trust Research Programme, Kilifi, PO Box 230, Kenya

**Keywords:** Child, Africa, Anaemia, haemoglobin measurement, sensitivity and specificity, inter-observer variation, prevalence, malaria endemic

## Abstract

**Background: **Severe anaemia in children requiring hospital admission is a major public health problem in malaria-endemic Africa. Affordable methods for the assessment of haemoglobin have not been validated against gold standard measures for identifying those with severe anaemia requiring a blood transfusion, despite this resource being in short supply.

**Methods: **We conducted a prospective descriptive study of hospitalized children aged 2 months – 12 years at Mbale and Soroti Regional Referral Hospitals, assessed to have pallor at triage by a nurse and two clinicians. Haemoglobin levels were measured using the HemoCue ^®^ Hb 301 system (gold standard); the Haemoglobin Colour Scale; Colorimetric and Sahli’s methods. We report clinical assessments of the degree of pallor, clinicians’ intention to transfuse, inter-observer agreement, limits of agreement using the Bland-Altman method, and the sensitivity and specificity of each method in comparison to HemoCue ^®^

**Results: **We recruited 322 children, clinically-assessed by the admitting nurse (n=314) as having severe (166; 51.6%), moderate (97; 30.1%) or mild (51; 15.8%) pallor. Agreement between the clinicians and the nurse were good: Clinician A Kappa=0.68 (0.60–0.76) and Clinician B Kappa=0.62 (0.53–0.71) respectively ( *P*<0.0001 for both). The nurse, clinicians A and B indicated that of 94/116 (81.0%), 83/121 (68.6%) and 93/120 (77.5%) respectively required transfusion. HemoCue ^®^ readings indicated anaemia as mild (Hb10.0–11.9g/dl) in 8/292 (2.7%), moderate (Hb5.0–9.9g/dl) in 132/292 (45.2%) and severe (Hb<5.0g/dl) in 152/292 (52.1%). Comparing to HemoCue® the Sahli’s method performed best in estimation of severe anaemia, with sensitivity 84.0% and specificity 87.9% and a Kappa score of  0.70 (0.64–0.80).

**Conclusions**: Clinical assessment of severe pallor results has a low specificity for the diagnosis of severe anaemia. To target blood transfusion Hb measurement by either Hemocue® or Sahli’s method for the cost of USD 4 or and USD 0.25 per test, respectively would be more cost-effective.

## Introduction

Anaemia is a major public health problem in low income and malaria endemic areas ^1, 2^. Its consequences vary with severity, duration, age, maternal parity and underlying cause. The World Health Organization (WHO) defines severe anaemia as a haemoglobin (Hb) concentration of <5.0g/dl (in the presence of malaria) or < 6g/dl which equates to a haematocrit of <15% or 18% respectively. In sub-Saharan Africa (sSA) there is a need to balance the demand for blood for transfusion with the scarcity of blood supply ^3^. Currently, there are insufficient data to be sure whether routinely giving blood to clinically stable children with a Hb of <5.0g/dl in malaria-endemic areas reduces immediate or long-term morbidity and mortality. As such, cost effective allocation of resources currently requires that only patients with symptomatic severe anaemia who have a high risk of a poor outcome should be transfused ^1, 4, 5^. Against this background, not all children with a Hb <5.0g/dl receive a blood transfusion ^6^, even though the WHO recommends it.

In resource-limited regions of sSA the choice of a diagnostic test is tagged to its sensitivity and specificity and weighed against its cost. Affordable and technologically appropriate methods are promoted for routine health care ^7^. Highly specific and sensitive tests are desirable for both clinical care and research purposes. For the assessment of anaemia, Hb estimation is more specific and more readily measurable than packed cell volume (PCV), and is also more pragmatic as it is less hazardous for health personnel ^8^. Despite being a common presentation and a leading cause of admissions in paediatric wards in Eastern Uganda, accurately testing for anaemia and its severity in the laboratory or at the bedside remains a challenge ^9, 10^. As a result, the diagnosis of anaemia and the decision to transfuse is often made clinically, through detection for the presence of pallor. Clinical assessment of pallor has been shown to be affected by wide inter-rater variability ^11^ and inaccuracy ^12^. However, most previous studies have been conducted in out-patients or in communities that have been aimed at assessing less severe degrees of anaemia. As a result, many African children are transfused who do not meet the WHO criteria for transfusion ^6^. In addition, laboratory estimation of Hb in East African district hospitals is among one of the least accurate tests ^13^.

The stimulus to carry out this study was to identify an accurate and reliable method for haemoglobin assessment that could contribute to rational decision making in resource-limited malaria endemic areas ^3^. We therefore aimed to compare the accuracy of four methods of haemoglobin estimation that are commonly available in Uganda in terms of their ability to correctly identify children who are clinically severely anaemic and require a blood transfusion: (i) Sahli’s method; (ii) Haemoglobin Colour Scale (HCS); (iii) Colorimetric method; and (iv) clinical assessment. The performance of each of these methods was compared to results obtained from the HemoCue® method, which we used, pragmatically, as our ‘gold standard’ for the purposes of this study ^14^.

## Methods

We conducted an observational study among children aged 2 months - 12 years who were admitted to the paediatric wards of Mbale and Soroti Regional Referral Hospitals (RRH) in Eastern Uganda. This is an area where the transmission of malaria is perennial, with peaks that correspond with the main rainy seasons. Concurrent with the timing of this study, an entomological inoculation rate (EIR) of 125 (95%CI: 72.2 – 183.0) was measured in Tororo district, 145km and 45km south of Soroti and Mbale respectively ^15^. Mbale RRH is a 470-bedded hospital with 95 paediatric beds while Soroti RRH has 274-beds in total including 64 for paediatrics. They receive, respectively, approximately 17,000 and 8,000 paediatric admissions per year. Both are staffed by paediatricians, medical officers, clinical officers and nursing staff and have simple functional side-room laboratories. At the time of this study, the methods used for transfusion decision making were clinician assessment and Sahli’s method. The Colorimetric method was being introduced in Mbale RRH and the Haemoglobin colour scale (HbCS) ^16^, was about to be introduced by the Uganda Ministry of Health. The HemoCue® equipment and reagents were provided through the Fluid Expansion as Supportive Therapy trial (FEAST; ISRCTN 69856593) ^17^.

### Clinical training and assessment

Pre-study training was conducted through which the admitting nurses and clinician at each centre were taught how to classify pallor. Three levels were taught: (i) mild (suggested to be synonymous with Hb level 10.0–11.9); (ii) moderate (estimated Hb 5.0–9.9g/dl); and (iii) severe (estimated Hb<5g/dl). Refresher training on eligibility criteria for transfusion was also covered. Briefly, the Uganda National Paediatric Guidelines indicate that a child should be only be transfused if their haemoglobin is <4g/dl or <6g/dl in the presence of life-threatening complications such as hypoxia with cardiac compensation, acidosis, dyspnoea, impaired consciousness, septicaemia, meningitis, cerebral malaria or hyperparasitaemia (>20%) ^18^.

At triage the admitting nurse, indicated the degree of clinical pallor (mild, moderate, severe) and whether, on the basis of this assessment, transfusion should be prescribed. A paediatric admission record (PAR) was completed by the admitting clinicians (Clinician A and B). All assessors were blind to the judgements of the others and whether the child should be transfused. Following these 3 clinical assessments, Hb levels were measured using the four laboratory methods ^19^. The ultimate decision on whether or not children should be transfused was based on the result from Sahli’s method, as this was the current practice in these hospitals. No specific delays to the receipt of treatment were introduced through the conduct of this study.

### Haemoglobin assessment methods

**Figure 1.**
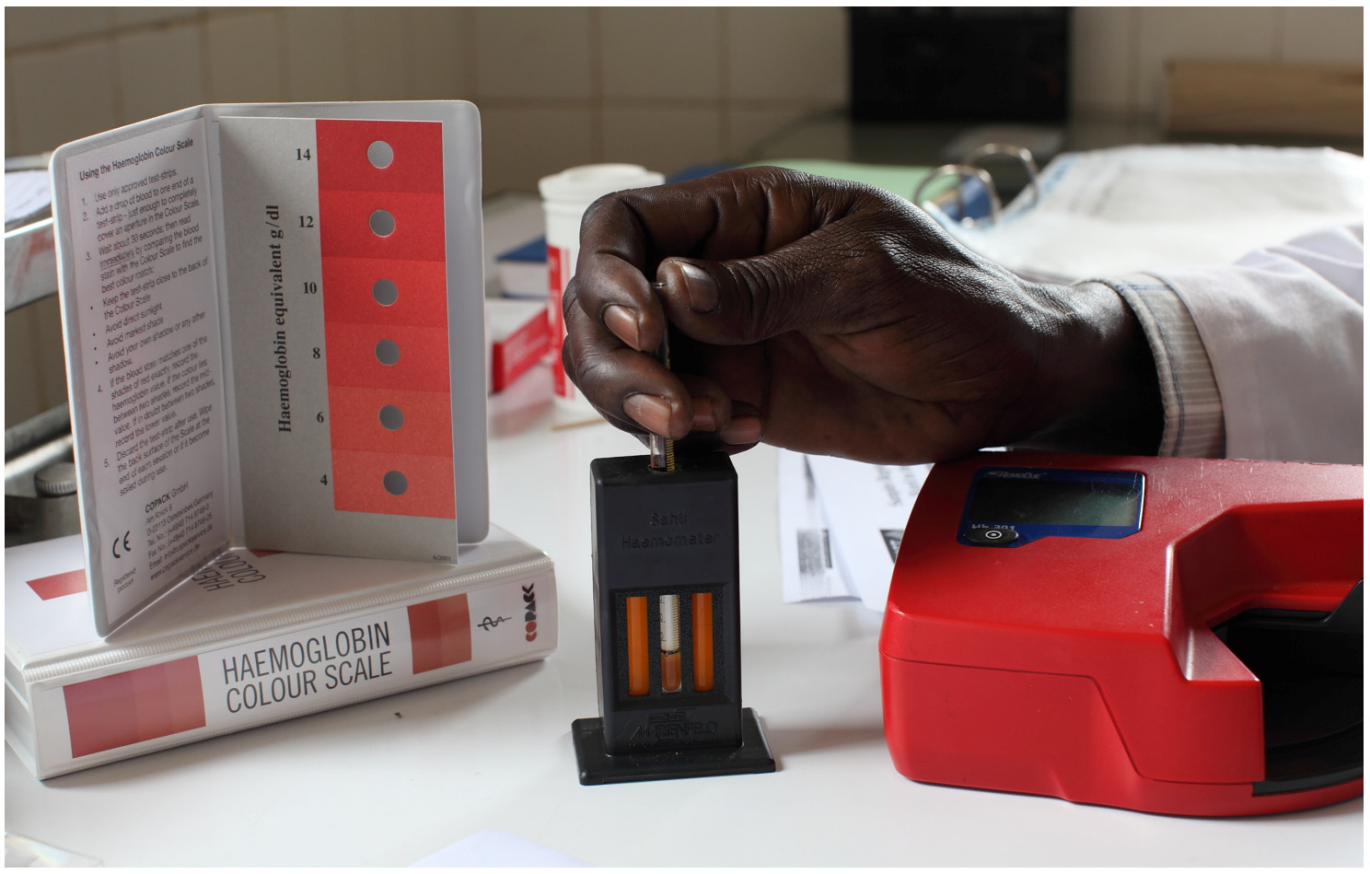
The Haemoglobin Colour Scale, Sahli’s meter and HemoCue® analyser for rapid assessment of haemoglobin status.

The three different methods for haemoglobin assessment are shown in Figure 1. The HemoCue® Hb 301 system (Hemocue AB, Angelholm, Sweden) is a portable point of care Hb testing device that uses disposable microcuvettes and requires no calibration. A finger-prick drop of blood is sucked into a disposable cuvette via capillary action before inserting into the Hemocue ^®^ machine which reads out the Hb value on a digital display within 15–45 seconds. The measurement range is 0–25.6g/dl. The Haemoglobin Colour Scale (HbCS) was developed by Department of Essential Health Technologies, World Health Organization (WHO), Geneva as an inexpensive, simple tool to measure haemoglobin levels in resource-poor settings to detect and control anaemia ^20^. It comprises of a card with six shades of red that represent Hb levels at 4, 6, 8, 10 12, and 14g/dl respectively. The test takes 30 seconds and the colour of the blood spot is matched against the hues on the kit’s colour scale. The scale does not include a colour corresponding to Hb of 5g/dl so all colour values of 4 and below were regarded as having Hb <5g/dl. The Colorimetric method is an accurate method for the estimation of Hb concentration as a continuous variable that involves conversion of Hb to cyanmethaemoglobin by potassium ferricyanide ^21^. The absorbance of the resultant solution is compared to the standard cyanmethaemoglobin solution by using a formula to obtain the amount of haemoglobin. The Colorimetric machine currently used is automated and gives the amount of Hb without manual calculations but takes about 10 minutes per test in addition to the time required to set up and calibrate the machine. Owing to the test being more time consuming, and reliant upon toxic cyanide reagents, it is less favoured for use in blood banks for Hb estimation. Finally, Sahli’s method uses a drop of blood pipetted into a graduated tube containing hydrochloric acid. The blood is converted to acid haematin which is then diluted with drops of distilled water until its colour matches the colour of the tinted comparator glass. The haemoglobin is estimated by reading the value directly from the scale on Sahli’s tube according to the height that the final solution has reached. This method must be read in an environment of natural light and takes only 2–3 minutes. The Hb level is read to one decimal point in grams per decilitre.

### Statistical analysis

All statistical analyses were performed using R statistical software (version 3.5.1 for Windows) and MedCalc v18 for Windows (version 18.2.1, MedCalc Software, OstendMariakerke, Belgium). We used HemoCue® as the gold standard in this study because of its proven sensitivity and specificity across a wide range of Hb values ^22^. The four other Hb estimation methods were tested against the HemoCue® Hb 301 system. We defined mild anaemia as Hb 10.0–11.9g/dl, moderate anaemia as Hb 5.0–9.9g/dl and severe anaemia as Hb <5.0g/dl. The diagnostic accuracy (specificity and sensitivity) of each method was calculated as previously described ^23^. The Bland Altman ^24^ approach was used to analyse the agreement between the HemoCue® and other methods. Briefly, this approach assumes that if two methods are to agree then the mean of the difference between every paired determination will not be statistically different from zero. By using this approach, we calculated limits of agreement (within a given confidence interval) between the two methods and graphically visualized the dispersion of these differences across the Hb values. Cohen’s kappa-statistic (κ) was used for assessing inter observer variation, through which agreement is scaled between zero and one, where 0 indicates no agreement (i.e. no better than would be observed by chance), and 1 signifies perfect agreement. Finally, we performed receiver-operating characteristic (ROC) curve analysis to examine the overall discriminatory power and calculability of the methods. Diagnostic performance for each method was ascertained from the area under the curve (AUC) for each method ^25^. 

The study was approved by the Mbale Regional Referral Hospital Research Ethics Committee (MRRH-REC) and the Uganda National Council of Science and Technology (UNCST).

## Results

### Participants

All children aged over 2 months to 12 years were assessed at triage for pallor. Children were included, following parental consent, if assessed to have a pallor. Children whose guardian or parent declined consent were excluded from the study. We recruited a total 322 participants: 200 (62.3%) from Mbale and 122 (37.7%) from Soroti RRHs. Enrolment was unselective, including all consecutive admissions between 8am and 5pm Monday to Friday during the period 2nd–15th February 2011. The demographic and clinical characteristics of study participants are outlined in Table 1. Overall, their median age [interquartile range (IQR)] was 29 (14–49) months; 38 (16.3%) were < 1 year; 155 (66.5%) were between 1–4 years and 40 (17.2%) 5 or more years of age. Although most were febrile on the basis of clinical history, less than half were pyrexial on measurement of axillary temperatures ( Table 1). The majority 276/286 (96.5%) had malaria confirmed on blood film examination. The median duration of admission was 3 (2–4) days.

**Table 1.** Demographic and clinical features of children with pallor in Eastern Uganda.

		N	Number of children with feature	%
**Demographic**				
	Sex	298		
	Male		168	56.4
	Age (Years)	233		
	< 5		193	82.8
	>5		40	17.2
**Symptoms**				
	Fever	312	311	99.7
	Cough	309	229	74.1
	Vomiting	308	208	67.5
	Diarrhoea	322	84	26.1
	Convulsions	322	30	9.3
	History of dark urine	197	37	18.8
**Signs**				
	Pyrexia (>37.5°C)	322	157	48.8
	Jaundice	302	177	58.6
	Increased respiratory rate	206	131	63.6
	Temperature Gradient	285	98	34.4
	CRT>2 seconds	322	129	40.1
	Weak pulse	268	27	10.1
	Sunken eyes	281	13	4.6
	Prostration	300	43	14.3
	Coma	322	86	26.7

Legend: N=number, %=percentage

### Clinical assessment of pallor and anaemia

The nurses assessed pallor as mild in 51 (15.8%), moderate in 97 (30.1%) and severe in 166 (51.6%) participants. Clinician A found mild pallor in 50 (15.7%) and Clinician B found mild pallor in 54 (17.0%) participants while, Clinicians A and B found severe pallor in 166 (51.9%) and 165 (51.9%) of study participants respectively. Whereas, the Hb gold standard laboratory measurement (HemoCue®) identified mild anaemia in 8(2.7%), moderate anaemia in 132 (45.2%) and severe anaemia in 152 (52.1%), respectively. Of participants with severe pallor, blood transfusion was recommended by the nurse for 94/152 (61.8%), by Clinician A in 83/212 (68.6%), and by Clinician B in 93/120 (77.5%) participants. On the basis of a HemoCue® reading, 152/292 (52.1 %) would have been eligible for transfusion.

### Comparing different diagnostic methods of Hb assessment

**Figure 2.**
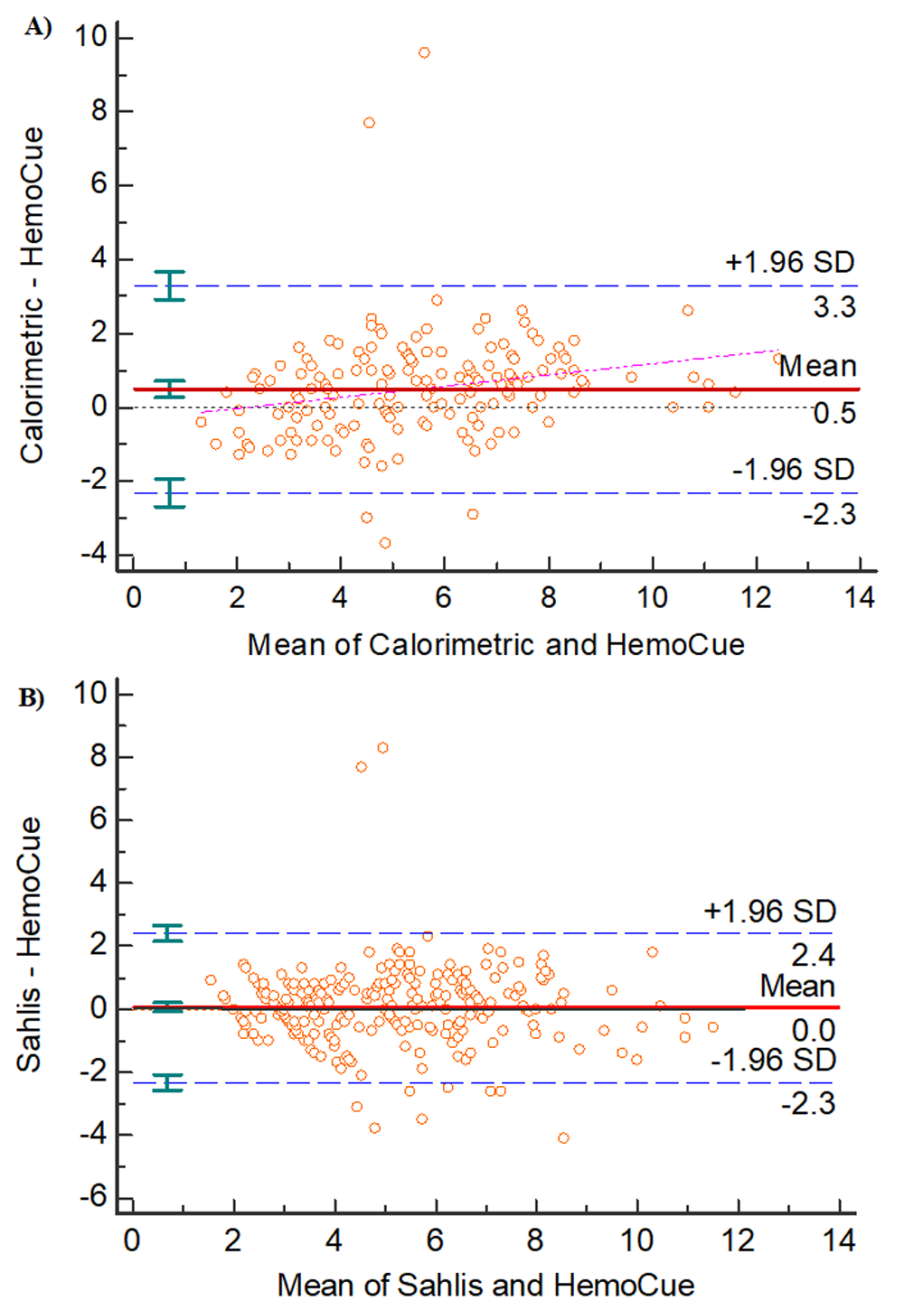
Bland-Altman plots for Colorimetric and Sahli’s methods compared to HemoCue as the reference. Bland-Altman plot of the individual differences between Hb values were plotted against the average Hb value as determined by pairs of Hb measurement methods. Each figure shows plots of the following differences and averages: **A**, HemoCue-Colorimetric; and **B**, HemoCue-Sahli’s. The red solid line indicates the null difference and the limits of agreement are shown by the blue dashed lines.

We first assessed the measurements of central tendency (mean and median) and variation (range and standard deviation, SD) for each of the Hb determining methods as shown in Table 2. We found that the HemoCue® method showed the lowest mean (5.0g/dl; SD=2.0) and the lowest median (4.8g/dl) when compared to both the Colorimetric and Sahli’s methods [mean=5.7g/dl (SD=2.5) and 5.1g/dl (SD=2.1)] respectively. The Bland-Altman concordance is shown in Figure 2: with the exception of outlying values, the majority of the measurement points were within 1.96 standard deviation of the mean paired differences. The mean difference with limits of agreement between HemoCue® and the Colorimetric method and between HemoCue® and Sahli’s method were 0.5 (95% CI -−12.3, 3.3) g/dl and 0.1 (95% CI- −2.3, 2.4) g/dl respectively ( Figure 1), implying good agreement between these methods, particularly for Sahli’s method.

**Table 2.** Measurements of central tendency and variation for the three methods of Hb determination.

Method	N	Mean ± SD ^a,^ ^b^	Median ^a^	Range (min; max ) ^a^
**HemoCue**	292	5.0 ± 2.0	4.8	0.7; 11.8
**Colorimetric**	188	5.7 ± 2.5	5.8	1.1; 13.8
**Sahli’s**	299	5.1 ± 2.1	5.0	1.8; 12.6

^a^Haemoglobin values in g/dl

^b^There were no statistically significant difference between the mean of the Hb determination for the three methods

^c^SD = Standard Deviation

### Reliability and validity of the various methods

**Figure 3.**
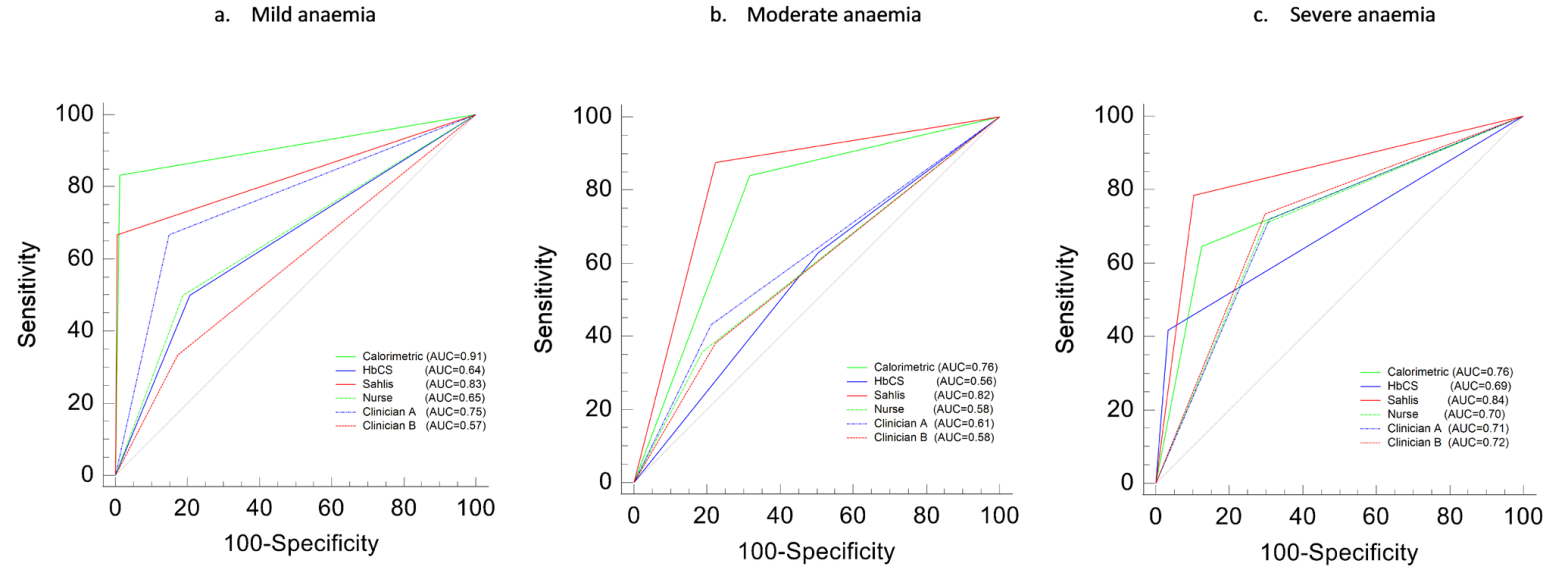
Receiver operating characteristic (ROC) curves for different methods used in the study. The figure shows the area under the receiver operator characteristic (ROC) curve for different Hb determining methods against HemoCue the gold standard with respect to three classifications of anaemia: **a**) mild, **b**) moderate, and **c**) severe. The area under the curve captures the relationship between the sensitivity and specificity of each method against the gold standard.

The sensitivities and specificities of each method in the diagnosis of mild, moderate and severe anaemia, compared to HemoCue® are summarised in Table 3 and Figure 3. Sahli’s method performed best, with sensitivity 84.0%, specificity 87.9% and positive and negative predictive values of 88.3% (82.6-92.3) and 83.6 (77.7-88.1) respectively. The corresponding values for the other methods are summarised in Table 3.

**Table 3.** Sensitivity, specificity, positive and negative predictive values of various methods of assessment of anaemia in children, using HemoCue as the gold standard.

Category	Method	Assessments	Prevalence N (%)	Sensitivity % (95% CI)	Specificity % (95% CI)	PPV % (95% CI)	NPV% (95% CI)
**Gold** **standard**	HemoCue®	Mild anaemia ^a^	8/292 (2.7)				
		Moderate anaemia ^b^	132/292 (45.2)				
		Severe anaemia ^c^	152/292 (52.1)				
**Clinical**	Nurse	Mild pallor ^a^	51/314 (15.8)	50.0 (15.7-84.3)	84.5 (79.8-88.5)	8.3 (4.1-16.1)	98.4 (96.8-99.2)
		Moderate pallor ^b^	97/314 (30.1)	40.6 (32.2-49.5)	78.6 (71.4-84.7)	61.4 (52.5-69.5)	61.3 (57.4-65.0)
		Severe pallor ^c^	166/314 (51.6)	70.8 (62.9-78.0)	70.2 (61.9-77.6)	71.8 (66.0-77.0)	69.2 (63.2-74.7)
	Clinician A	Mild pallor ^a^	50/318 (15.7)	62.5 (24.5-85.9)	85.9 (81.3-89.7)	11.1 (6.4-18.7)	98.0 (97.1-99.5)
		Moderate pallor ^b^	102/318 (32.4)	45.1 (36.5-54.0)	79.2 (72.1-89.3)	64.5 (56.0-72.2)	63.3 (59.2-67.2)
		Severe pallor ^c^	166/318 (51.9)	72.8 (65.0-79.8)	71.6 (63.4-78.9)	73.3 (67.5-78.4)	71.1 (65.0-76.5)
	Clinician B	Mild pallor ^a^	54/318 (17.0)	25.0 (3.2-65.1)	82.7 (77.8-87.0)	3.9 (1.2-12.2)	97.5 (96.3-98.4)
		Moderate pallor ^b^	99/318 (31.1)	39.8 (31.5-48.7)	79.2 (72.1-85.3)	61.6 (52.6-69.9)	61.2 (57.3-64.9)
		Severe pallor ^c^	165/318 (51.9)	73.5 (65.7-80.4)	70.9 (62.7-78.3)	73.0 (67.3-78.1)	71.4 (65.3-76.9)
**Laboratory**	Colorimetric	Mild anaemia ^a^	7/188 (3.7)	83.3 (35.9-99.6)	98.8 (95.7-99.9)	71.4 (37.6-91.2)	99.4 (96.5-99.9)
		Moderate anaemia ^b^	103/188 (54.8)	83.9 (74.1-91.2)	69.5 (59.1-78.7)	70.8 (63.7-77.0)	83.1 (74.6-89.2)
		Severe anaemia ^c^	75/188 (39.9)	66.3 (55.3-76.1)	87.3 (78.5-93.5)	83.8 (74.5-90.2)	72.4 (66.8-78.1)
	Sahli’s	Mild anaemia ^a^	6/299 (2.0)	50.0 (15.7-84.3)	99.6 (97.9-100)	80.0 (33.4-97.0)	98.5 (97.1-99.3)
		Moderate anaemia ^b^	143/299 (47.8)	86.4 (79.1-91.9)	82.2 (75.2-88.0)	80.0 (73.8-85.0)	88.0 (82.4-92.0)
		Severe anaemia ^c^	149/299 (49.8)	84.0 (77.0-89.6)	87.9 (81.2-93.0)	88.3 (82.6-92.3)	83.6 (77.7-88.1)
**Bedside**	HB color scale	Mild anaemia ^a^	46/263 (17.5)	37.5 (8.5-75.5)	82.9 (77.7-87.4)	6.5 (2.7-15.1)	97.7 (96.1-98.6)
		Moderate anaemia ^b^	130/263 (49.4)	51.1 (42.2-60.1)	50.3 (41.5-59.2)	50.4 (44.4-56.4)	51.2 (45.1-57.2)
		Severe anaemia ^c^	60/263 (22.8)	43.1 (34.2-52.3)	95.6 (90.7-98.4)	89.8 (79.7-95.2)	65.2 (61.5-68.7)

^a^Hb 10 – 11.9g/dl; ^b^Hb 5 – 9.9; ^c^Hb<5g/dl; PPV= Positive Predictive value; NPV= Negative Predictive value

The diagnostic performance of Sahli’s method in the assessment of severe anaemia as determined by AUC was 84%, superior to that of any of the other three methods ( Figure 2). The AUC values for the Colorimetric and HbCS methods were 75%, and 69%, respectively. The AUC values for the clinicians’ interpretation were 70% (nurse), 71% (Clinician A), and 72% (Clinician B). The Cohen’s Kappa statistic (κ) indicators comparing these three methods against the gold standard are summarised in Table 4. The HbCS rated very poorly for each of the three categories of anaemia: mild (κ=0.07), moderate (0.07) and severe (0.37). There was substantial agreement between the HemoCue® and Colorimetric method in the identification of mild anaemia, (κ=0.76, 0.50–1.00), but agreement was lower for severe anaemia (0.54; 0.41-0.66). Compared with HemoCue the Sahli’s method performed best across all categories including the identification of severe anaemia cases (κ=0.70, 0.64–0.80). Other methods, achieved fair-moderate reliability ( Table 4) but the agreement of clinical assessment (nurse and clinician) rated as poor despite the inter-observer agreement between the nurse and each of the clinicians being fairly good κ>0.60 ( Table 5).

**Table 4.** Kappa (κ) statistics for agreement of the various methods against the HemoCue gold standard.

Methods	Mild anaemia κ statistic (95% CI)	Moderate anaemia κ statistic (95% CI)	Severe anaemia κ statistic (95% CI)
**Colorimetric**	0.76 ( 0.50-1.00)	0.53 ( 0.40-0.65)	0.54 ( 0.41-0.66)
**HBCS**	0.07 ( 0.03-0.18)	0.07 ( 0.04-0.20)	0.37 ( 0.27-0.47)
**Sahli’s **	0.61 ( 0.29-0.92)	0.68 ( 0.60-0.76)	0.70 ( 0.64-0.80)
**Nurse**	0.10 ( 0.06-0.21)	0.19 ( 0.08-0.30)	0.41 ( 0.30-0.51)
**Clinician A**	0.14 ( 0.03-0.28)	0.24 ( 0.14-0.35)	0.44 ( 0.34-0.55)
**Clinician B**	0.04 ( 0.02-0.150	0.19 ( 0.09-0.30)	0.43 ( 0.34-0.54)

**Table 5.** Inter observer agreement between Nurse and Clinicians and Clinician A to Clinician B on severe pallor.

	Clinician A		Clinician B	
	Agreement	Kappa (95% CI)	Agreement	Kappa (95% CI)
**Nurse**	81.4	0.68 (0.60-0.76)	77.0	0.62 (0.53-0.71)
**Clinician A**			75.7	0.60 (0.51-0.69)

## Discussion

We investigated the diagnostic accuracy of a range of methods in the assessment of Hb values among children admitted to hospital in a malaria endemic area in Eastern Uganda. We found a higher concordance between assessors in the identification of clinical pallor than observed in previous studies ^26^. This may be explained by the joint training sessions that we held both during the FEAST trial and prior to this study ^27^. Nevertheless, on clinical assessment of pallor alone, both clinicians and nurses recommended transfusion in 62–78% of children, considerably higher than the rate that would have been justified by HemoCue® (52%). In comparison to HemoCue®, HbCS and the Colorimetric method rated very poorly in the identification of severe anaemia. There was, however, substantial agreement between the HemoCue® and Sahli’s method. Sahli’s method detected 6/321 (1.9%) children with severe anaemia in comparison to 9/322 (2.8%) by HemoCue®, and was the test that was most specific and sensitive. For mild anaemia, the Colorimetric test was the most sensitive test and the most specific test was the Colorimetric method.

As expected, blood transfusion was recommended in a high proportion of children on the basis of clinical assessment. Firstly, many children 131/206 (63.6%) presented with fast breathing, and it is possible that these were classified as respiratory distress, a danger sign requiring transfusion ^28^. In addition, a number of children had features of impaired circulation 129/322 (40.1%), one of the defining clinical characteristics for shock, coma 86/322 (26.7%) or prostration 43/300 (14.3%), all of which are indications for blood transfusion in anaemic patients in Uganda National Guidelines. Furthermore, in agreement with a study conducted in Pakistan ^26^, we found that clinical assessment was inaccurate in the diagnosis of mild and moderate anaemia. Previous studies have largely been conducted in outpatient settings or conducted outside Africa ^12, 29^ and, to the best of our knowledge, this is the first report conducted in African children presenting for admission with severe and life-threatening conditions.

HemoCue® has been shown to have reliable accuracy in determining Hb across the range of severity of anaemia ^14^. Nevertheless, few studies have included groups of children with profound anaemia (haemoglobin < 4g/dl) and therefore future research should focus on validation in this group. In keeping with previous studies, we found that the method was simple to operate, and allowed for Hb estimation at the bedside to give results within <30 seconds ^22^, allowing the attending clinicians to make rapid treatment decisions. Current costs for the two most efficient tests (in terms of positive and negative predictive values) approximately USD 4 per test for HemoCue® (after an initial cost of between USD 250-350 for the analyser) and USD 0.25 per test for Sahli’s method (after an initial cost of USD 40-50 for the meter). Of note however, is that HemoCue® can be performed at the bedside whereas Sahli’s method requires a functioning laboratory. Other limitations of Sahli’s method include (i) its accuracy being dependent on natural light, (ii) difficulty in attaining a uniform colour in the test tube; (iii) the reagents are unstable and deteriorate with storage; (iv) dilutional discrepancies can occur. Nevertheless, if laboratory technicians are properly trained this could offer an affordable and reliable method for accurately assessing Hb level. 

Our study had some limitations since sensitivity and specificity could not tell the probability of an individual patient having severe anaemia, a feature that would be useful for clinicians. We therefore included an analysis of positive and negative predictive that would give the clinician a better indication to rule in or rule out the need for a transfusion. The results of this study should, however, be interpreted with caution for children with severe anaemia in non-malaria endemic areas, where they should be validated in additional studies.

In conclusion, our data suggests that in situations where HemoCue® is either unaffordable or unavailable, among the commonly available methods, Sahli’s method provided the next best alternative for the detecting severe anaemia, and could be used to guide treatment and avoid over-use of blood transfusion in resource-limited settings.

## Declarations

### Data availability

The data underlying this study is available from Harvard Dataverse. Dataset 1: Evaluation of the diagnostic accuracy and cost of different methods for the assessment of severe anaemia in hospitalised children in Eastern Uganda- Dataset https://doi.org/10.7910/DVN/VMNDPO ^30^

This dataset is available under the terms of the Creative Commons Zero “No rights reserved” data waiver (CC0 1.0 Public domain dedication).

## Ethics approval and consent to participate

The Mbale Regional Referral Hospital Research & Ethics Committee (MRRH-REC) approved the study (RECIRC 109/2010), and local permission to conduct the study was obtained from both Mbale and Soroti Regional Referral Hospitals.

## Consent for publication

The Mbale Clinical Research Institute (MCRI, www.mcri.ac.ug), a research entity affiliated to the Uganda National Health Research Organization (UNHRO), permits the publication of this manuscript.
