# Supplementary material for: Evaluation of the diagnostic accuracy and cost of different methods for the assessment of severe  anaemia in hospitalised children in Eastern Uganda
Source: Wellcome Open Res. 2019 Mar 18;3:130. Originally published 2018 Oct 4. [Version 2] doi: 10.12688/wellcomeopenres.14801.2 (PMC6402076; doi:10.12688/wellcomeopenres.14801.2)
Supplement: Peer review history

# Peer review: Reviewer response for version 2

**DOI:** 10.21956/wellcomeopenres.16570.r35087

**Published:** 2019-03-19

**Authors:** Jean‐Pierre Allain

**Affiliations:** 1 Department of Haematology, University of Cambridge, Cambridge, UK

The revised manuscript has taken positively the remarks of this reviewer. The revised manuscript is suitable for indexing.

I have read this submission. I believe that I have an appropriate level of expertise to confirm that it is of an acceptable scientific standard.

# Peer review: Reviewer response for version 1

**DOI:** 10.21956/wellcomeopenres.16129.r34909

**Published:** 2019-03-05

**Authors:** Walter H Dzik

**Affiliations:** 1 Department of Pathology (Transfusion), Harvard University/Massachusetts General Hospital, Boston, Massachusetts, USA

This is a well conducted and well-written report on a topic of practical importance to many children in sub-Saharan Africa. 

 The findings might not be generalizable because of the effect of local training with laboratory techniques, but I believe the authors adequately acknowledge this. 

 I have one suggestion. The hemocue device (Hb 301) is the 'gold standard' claimed by the authors and all comparisons are pegged to its performance. For that reason, can the authors please cite literature that reports the degree of accuracy and the precision (variability) of this device at hemoglobin concentrations in the 3-5 g/dL range?

I have read this submission. I believe that I have an appropriate level of expertise to confirm that it is of an acceptable scientific standard.

# Response

**Published:** 2019-03-11

**Authors:** Kathryn Maitland

**Affiliations:** KEMRI-Wellcome Trust Research Programme, Kenya

The Hemocue device (Hb 301) is the 'gold standard' claimed by the authors and all comparisons are pegged to its performance. For that reason, can the authors please cite literature that reports the degree of accuracy and the precision (variability) of this device at hemoglobin concentrations in the 3-5 g/dL range?

*Whilst the HemoCue has a measuring range: 0 - 25.6 g/dl we agree that it has not been comprehensively evaluated for children with haemoglobins < 4g/dl. The closest study included in the references is Evaluation and costs of different haemoglobin methods for use in district hospitals in Malawi Medina et al.  We have added a couple of sentences to make this point and suggest this should be an area for future investigation.*

# Peer review: Reviewer response for version 1

**DOI:** 10.21956/wellcomeopenres.16129.r34908

**Published:** 2019-02-19

**Authors:** Jean‐Pierre Allain

**Affiliations:** 1 Department of Haematology, University of Cambridge, Cambridge, UK

The manuscript by Olupot-Olupot reports a comparison of clinical and Hb assay estimation of anaemia severity in two hospitals in Uganda. It concludes that the Sahli’s method is accurate and cheap to identify patients with Hb levels <5g/dl eligible for transfusion.

1. Throughout the manuscript there is a persistent misspelling of the colorimetric method (properly spelled at beginning of page 4) for ‘calorimetric’. The test having to do with colour, not heat, so colorimetric should be used unless the instrument and presumably the registered system is indeed labelled ‘calorimetric’.
2. The description of the ‘Colour Scale’ method against the presented Figure 1 is unclear. The scale bands in the Figure are presenting an empty circle that might suggest a space to deposit blood in order to directly compare with the printed scale. If not, what is the use of this circle? If not, on what support is a drop of blood deposited to compare to the scale?
3. In Table 1, there is a considerable deficit in age recording. Why?
4. The main objective of the Hb estimation is decision-making regarding indication of transfusion. In Table 3, it appears that the percentage of severe pallor, irrespective of the observer, is very close to the percentage scored by HemoCue. It would be interesting to provide a Table correlating the clinicians’ decision to transfuse with their estimate of pallor. Taking the 152 cases of severe anaemia identified with the gold standard as <5g/dl and eligible for transfusion according to WHO, how many of those were correctly identified as severe pallor by clinicians and how many were considered for transfusion? In addition, it would be useful to know the reasons why X number of moderate or mild pallor patients was identified by clinicians eligible for transfusion.

I have read this submission. I believe that I have an appropriate level of expertise to confirm that it is of an acceptable scientific standard, however I have significant reservations, as outlined above.

# Response

**Published:** 2019-03-11

**Authors:** Kathryn Maitland

**Affiliations:** KEMRI-Wellcome Trust Research Programme, Kenya

1. Throughout the manuscript there is a persistent misspelling of the colorimetric method (properly spelled at beginning of page 4) for ‘calorimetric’. The test having to do with colour, not heat, so colorimetric should be used unless the instrument and presumably the registered system is indeed labelled ‘calorimetric’. *Response:*

*Thank you for pointing this out; this must have happened during a final spellcheck replacing the original with calorimetric, we have corrected this.*

2.The description of the ‘Colour Scale’ method against the presented Figure 1 is unclear. The scale bands in the Figure are presenting an empty circle that might suggest a space to deposit blood in order to directly compare with the printed scale. If not, what is the use of this circle? If not, on what support is a drop of blood deposited to compare to the scale?

*The Figure is only a picture of all three devices for measurement side by side. We are sorry that this was a little confusing. As explained in the text under the methods section for the Colour Scale ‘* *It (the* *Colour Scale)* *comprises of a card with six shades of red that represent Hb levels at 4, 6, 8, 10 12, and 14g/dl respectively. The test takes 30 seconds and the colour of the blood spot is matched against the hues on the kit’s colour scale.* *The intention is not for the blood to be spotted onto the scale.*

3. In Table 1, there is a considerable deficit in age recording. Why?

*The purpose of the age groups presented < 5 years and > 5 years was to provide overarching summaries; since we do not stratify the results by age group. However we are happy to split down the age groups further: we have provided further breakdown for children < 1 year; children 1-4 years and those > 5 years.*

*We have added these into the results section.*

4. The main objective of the Hb estimation is decision-making regarding indication of transfusion. In Table 3, it appears that the percentage of severe pallor, irrespective of the observer, is very close to the percentage scored by HemoCue. It would be interesting to provide a Table correlating the clinicians’ decision to transfuse with their estimate of pallor. Taking the 152 cases of severe anaemia identified with the gold standard as <5g/dl and eligible for transfusion according to WHO, how many of those were correctly identified as severe pallor by clinicians and how many were considered for transfusion? In addition, it would be useful to know the reasons why X number of moderate or mild pallor patients was identified by clinicians eligible for transfusion. *C*

*Children were all assessed clinically before blood was tested by each of the methods. The specificity of assessment for severe pallor for accurately predicting severe anaemia ranged from* *70.2% (61.9-77.6) to 71.6 (63.4-78.9) and the positive predictive value was 71.8 (66.0-77.0) to 73.0 (67.3-78.1); so the correlations are not as close as indicated.* *We agree that it is confusing that even after pre-study training doctors and nurses indicated their desire to transfuse. One reason is probably because the evidence supporting current guidelines are only based on expert opinion and there have been quite a number of reports (indicated in the manuscript) where doctors are not following national or international guidelines and transfusing at much higher thresholds.*

# Peer review: Reviewer response for version 1

**DOI:** 10.21956/wellcomeopenres.16129.r34130

**Published:** 2018-11-14

**Authors:** Dora Mbanya

**Affiliations:** 1 Department of Hematology, University of Yaoundé I, Yaoundé, Cameroon

This is an interesting paper with a pertinent topic, of great relevance to resource-limited settings especially of sub-Saharan Africa.

 A lot of blood is consumed in these regions, where anaemia is aggravated by holoendemic malaria especially in the Paediatrics and Obstetrics/Gynaecology Units, and despite the low donation/collection rates.

 Furthermore, the topic addresses costs, which are a limiting factor to appropriate care in resource-limited settings.

 In evaluating the diagnostic accuracy and cost of different haemoglobin-estimation techniques, the authors have selected low-cost techniques and compared their performance to a low-cost “gold standard”, which is very appropriate.

 Appropriate statistical methods have been used in data analysis and the findings seem acceptable. However, there are a few general remarks:

1.  A few grammatical errors to correct (example: use of the wrong tense – “ **used**” instead of “ **use**” etc.)
2. ***The Abstract*** presents inconsistent results, and the arithmetics must be corrected: The total of 164 severely anaemic children, 99 moderate cases and 57 mild cases sums up to 320 children and not 322. Similarly, their corresponding percentages (51%, 30.7% and 17.7%) only add up to 99.4% and not 100%.
3. ***T*** *he Introduction *also talks of “Table 3” as the first table of the paper. Tables and Figures should be introduced in chronological order. Hence, this, being the first table introduced in the article, should be “Table 1”.
4. ***The Results: ***  children under 12 years do not give direct consent. If guardians are providing consent, then, “ **assent**” should be used. Furthermore, that information should be under METHODS and not under RESULTS.
5. As mentioned above, Table 1 should now read Table 2 and Table 2 should now read Table 3 etc. etc.
6. ***The Conclusions*** respond to the research question.

I have read this submission. I believe that I have an appropriate level of expertise to confirm that it is of an acceptable scientific standard.

# Response

**Published:** 2019-03-11

**Authors:** Kathryn Maitland

**Affiliations:** KEMRI-Wellcome Trust Research Programme, Kenya

1. A few grammatical errors to correct (example: use of the wrong tense – “used ” instead of “use ”etc.)

*We have spelled checked and corrected these*

2. The Abstract presents inconsistent results, and the arithmetics must be corrected: The total of 164 severely anaemic children, 99 moderate cases and 57 mild cases sums up to 320 children and not 322. Similarly, their corresponding percentages (51%, 30.7% and 17.7%) only add up to 99.4% and not 100%.

*Thank you for noting this. We corrected these numbers.*

3.The Introduction  also talks of “Table 3” as the first table of the paper. Tables and Figures should be introduced in chronological order. Hence, this, being the first table introduced in the article, should be “Table 1”.

*We agree that this is not relevant in the methods section so we have removed the reference to Table 3 results at this point. We therefore do not require the production team to reorder the tables.*

4. The Results: children under 12 years do not give direct consent. If guardians are providing consent, then, “assent ” should be used. Furthermore, that information should be under METHODS and not under RESULTS.

*We have added the word ‘parental’ into the consent sentence. But believe that the following sentence in the original manuscript did qualify this was parental consent. We are sorry this was misleading.*

**

*‘Children were included, following parental consent, if assessed to have a pallor. Children whose guardian or parent declined consent were excluded from the study.’*

5. As mentioned above, Table 1 should now read Table 2 and Table 2 should now read Table 3 etc.etc.

*We request the production team to remove the reference to Table 3 in the methods therefore reordering the tables in not required.*

6. The Conclusions respond to the research question.

*We have reread the final conclusion and believe our summary reflects on the question we posed: which method would reliably assess haemoglobin.*
